# 1-Allyl-3-methyl-3′,5′-diphenyl­spiro­[quinoxaline-2(1*H*),2′(3′*H*)-[1,3,4]thia­diazole]

**DOI:** 10.1107/S1600536810046994

**Published:** 2010-11-20

**Authors:** Caleb Ahoya Anothane, Rachid Bouhfid, Hafid Zouihri, El Mokhtar Essassi, Seik Weng Ng

**Affiliations:** a Laboratoire de Chimie Organique Hétérocyclique, Pôle de Compétences Pharmacochimie, Université Mohammed V-Agdal, BP 1014 Avenue Ibn Batout, Rabat, Morocco; b Department of Chemistry, University of Malaya, 50603 Kuala Lumpur, Malaysia

## Abstract

In the title spiro compound, C_25_H_22_N_4_S, the planar quinoxaline (r.m.s. deviation = 0.070 Å) and planar thia­diazole (r.m.s. deviation = 0.060 Å) ring systems share a common C atom; their mean planes are aligned at 89.7 (1)°. The thia­zole ring possesses two aromatic ring substituents and is nearly coplanar with these rings [the dihedral angles between the thia­diazole and phenyl rings are 5.7 (1) and 10.7 (1)°]. The allyl unit is disordered over two positions in a 0.65 (1):0.35 (1) ratio.

## Related literature

For pharmacologically active compounds derived from the 1,3-dipolar addition of diphenyl­nitrilimine to double bonds, see: Ahabchane & Essassi (2000 ▶); Canara *et al.* (2004 ▶); Ghomsi *et al.* (2004 ▶); Mustaphil *et al.* (2005 ▶).
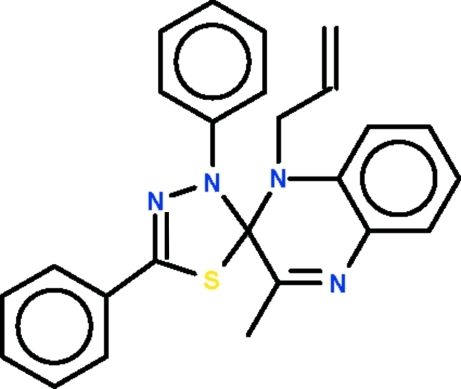


## Experimental

### 

#### Crystal data

C_25_H_22_N_4_S
*M* *_r_* = 410.53
Triclinic, 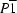

*a* = 7.9201 (1) Å
*b* = 10.0713 (1) Å
*c* = 13.6642 (2) Å
α = 78.296 (1)°
β = 81.277 (1)°
γ = 89.826 (1)°
*V* = 1054.50 (2) Å^3^
*Z* = 2
Mo *K*α radiation
μ = 0.17 mm^−1^
*T* = 293 K
0.40 × 0.10 × 0.10 mm

#### Data collection

Bruker X8 APEXII diffractometer
Absorption correction: multi-scan (*SADABS*; Sheldrick, 1996 ▶) *T*_min_ = 0.818, *T*_max_ = 0.862
38334 measured reflections
8514 independent reflections
5771 reflections with *I* > 2σ(*I*)
*R*_int_ = 0.022

#### Refinement

*R*[*F* ^2^ > 2σ(*F* ^2^)] = 0.050
*wR*(*F* ^2^) = 0.164
*S* = 1.03
8514 reflections
291 parameters
28 restraints
H-atom parameters constrained
Δρ_max_ = 0.32 e Å^−3^
Δρ_min_ = −0.20 e Å^−3^

### 

Data collection: *APEX2* (Bruker, 2007 ▶); cell refinement: *SAINT* (Bruker, 2007 ▶); data reduction: *SAINT*; program(s) used to solve structure: *SHELXS97* (Sheldrick, 2008 ▶); program(s) used to refine structure: *SHELXL97* (Sheldrick, 2008 ▶); molecular graphics: *X-SEED* (Barbour, 2001 ▶); software used to prepare material for publication: *publCIF* (Westrip, 2010 ▶).

## Supplementary Material

Crystal structure: contains datablocks global, I. DOI: 10.1107/S1600536810046994/nk2073sup1.cif

Structure factors: contains datablocks I. DOI: 10.1107/S1600536810046994/nk2073Isup2.hkl

Additional supplementary materials:  crystallographic information; 3D view; checkCIF report

## Figures and Tables

**Fig. 1.**
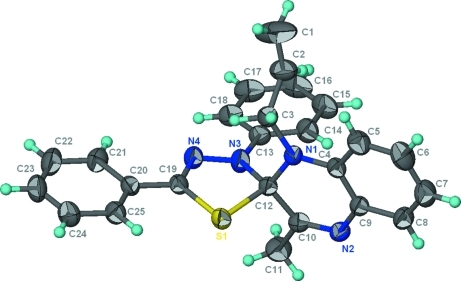
Displacement ellipsoid plot of C25H22N4S at the 50% probability level; hydrogen atoms are drawn as arbitrary radius. The minor disorder component is not shown.

## supplementary crystallographic information

### Comment

In our studies on pharmacologically active compounds, we have used diphenylnitrilimine to undergo 1,3-dipolar additions to double bonds (Ahabchane & Essassi, 2000; Canara *et al.*, 2004; Ghomsi *et al.*, 2004; Mustaphil *et al.*, 2005). In the present study, this compound reacts with an aromatic thione to yield the title spiro compound (Scheme I, Fig. 1). The quinoxaline and the thiadiazole ring systems share a common C atom; their mean planes are aligned at 89.7 (1)°.

### Experimental

To a solution of 1-allyl-3-methylquinoxaline-2-thione (1.00 g, 4.62 mmol) and diphenylnitrilimine (1.28 g, 5.55 mmol) in THF (20 ml) was added triethylamine (0.78 ml. 5.55 mmol). The mixture was heated for 24 h. The precipitate was recovered by filtration and was separated by chromatography on silica gel (hexane/ethyl acetate: 9/1). The title compound was obtained as yellow crystals upon evaporation of the solvent.

### Refinement

C-bound H-atoms were placed in calculated positions (C—H 0.93–0.97 Å) and were included in the refinement in the riding model approximation, with *U*(H) set to 1.2–1.5*U*_eq_(C). The allyl unit is disordered over two positions in a 65 (1):35 (1) ratio.

### Crystal data

**Table:**

C_25_H_22_N_4_S	*Z* = 2
*M**_r_* = 410.53	*F*(000) = 432
Triclinic, *P*1	*D*_x_ = 1.293 Mg m^−^^3^
Hall symbol: -P 1	Mo *K*α radiation, λ = 0.71073 Å
*a* = 7.9201 (1) Å	Cell parameters from 9958 reflections
*b* = 10.0713 (1) Å	θ = 2.3–33.0°
*c* = 13.6642 (2) Å	µ = 0.17 mm^−^^1^
α = 78.296 (1)°	*T* = 293 K
β = 81.277 (1)°	Prism, yellow
γ = 89.826 (1)°	0.40 × 0.10 × 0.10 mm
*V* = 1054.50 (2) Å^3^	

### Data collection

**Table:**

Bruker X8 APEXII diffractometer	8514 independent reflections
Radiation source: fine-focus sealed tube	5771 reflections with *I* > 2σ(*I*)
graphite	*R*_int_ = 0.022
φ and ω scans	θ_max_ = 33.9°, θ_min_ = 2.1°
Absorption correction: multi-scan (*SADABS*; Sheldrick, 1996)	*h* = −12→12
*T*_min_ = 0.818, *T*_max_ = 0.862	*k* = −15→15
38334 measured reflections	*l* = −21→21

### Refinement

**Table:**

Refinement on *F*^2^	Primary atom site location: structure-invariant direct methods
Least-squares matrix: full	Secondary atom site location: difference Fourier map
*R*[*F*^2^ > 2σ(*F*^2^)] = 0.050	Hydrogen site location: inferred from neighbouring sites
*wR*(*F*^2^) = 0.164	H-atom parameters constrained
*S* = 1.03	*w* = 1/[σ^2^(*F*_o_^2^) + (0.0836*P*)^2^ + 0.1531*P*] where *P* = (*F*_o_^2^ + 2*F*_c_^2^)/3
8514 reflections	(Δ/σ)_max_ = 0.001
291 parameters	Δρ_max_ = 0.32 e Å^−^^3^
28 restraints	Δρ_min_ = −0.20 e Å^−^^3^

### Fractional atomic coordinates and isotropic or equivalent isotropic displacement parameters (Å^2^)

**Table:**

	*x*	*y*	*z*	*U*_iso_*/*U*_eq_	Occ. (<1)
S1	0.55728 (4)	0.85155 (3)	0.59838 (2)	0.04746 (11)	
N1	0.50074 (13)	0.71267 (12)	0.79755 (8)	0.0449 (2)	
N2	0.15933 (13)	0.76834 (10)	0.77459 (8)	0.0433 (2)	
N3	0.50993 (16)	0.59101 (10)	0.66315 (9)	0.0507 (3)	
N4	0.64611 (15)	0.60663 (10)	0.58661 (9)	0.0470 (2)	
C1	0.8280 (5)	0.5283 (5)	0.9283 (4)	0.0709 (12)	0.654 (11)
H1A	0.9040	0.5982	0.9290	0.085*	0.654 (11)
H1B	0.8382	0.4425	0.9674	0.085*	0.654 (11)
C2	0.7059 (6)	0.5510 (3)	0.8718 (4)	0.0527 (9)	0.654 (11)
H2	0.6314	0.4796	0.8723	0.063*	0.654 (11)
C1'	0.8401 (10)	0.5022 (8)	0.8777 (11)	0.085 (3)	0.346 (11)
H1'1	0.9090	0.5189	0.8148	0.102*	0.346 (11)
H1'2	0.8650	0.4320	0.9287	0.102*	0.346 (11)
C2'	0.7060 (13)	0.5787 (8)	0.8948 (4)	0.0579 (19)	0.346 (11)
H2'	0.6338	0.5656	0.9565	0.070*	0.346 (11)
C3	0.68125 (17)	0.68768 (17)	0.80623 (11)	0.0584 (4)	
H3A	0.7262	0.7583	0.8348	0.070*	
H3B	0.7456	0.6924	0.7393	0.070*	
C4	0.39793 (16)	0.76799 (11)	0.86969 (9)	0.0406 (2)	
C5	0.4548 (2)	0.79361 (17)	0.95621 (12)	0.0577 (3)	
H5	0.5670	0.7761	0.9662	0.069*	
C6	0.3444 (3)	0.84499 (19)	1.02685 (13)	0.0684 (4)	
H6	0.3840	0.8623	1.0839	0.082*	
C7	0.1772 (3)	0.87124 (17)	1.01491 (12)	0.0649 (4)	
H7	0.1043	0.9053	1.0634	0.078*	
C8	0.11982 (19)	0.84633 (14)	0.93028 (11)	0.0527 (3)	
H8	0.0071	0.8637	0.9216	0.063*	
C9	0.22789 (15)	0.79542 (11)	0.85726 (9)	0.0400 (2)	
C10	0.25755 (16)	0.72698 (12)	0.70458 (10)	0.0432 (2)	
C11	0.1835 (2)	0.69551 (19)	0.61697 (13)	0.0644 (4)	
H11A	0.0655	0.7193	0.6226	0.097*	
H11B	0.1924	0.6004	0.6172	0.097*	
H11C	0.2452	0.7467	0.5549	0.097*	
C12	0.44934 (15)	0.71136 (11)	0.70212 (9)	0.0396 (2)	
C13	0.44801 (17)	0.45774 (11)	0.70791 (10)	0.0446 (3)	
C14	0.3295 (2)	0.43079 (15)	0.79643 (12)	0.0581 (4)	
H14	0.2914	0.5007	0.8287	0.070*	
C15	0.2686 (2)	0.29836 (16)	0.83610 (14)	0.0659 (4)	
H15	0.1879	0.2807	0.8945	0.079*	
C16	0.3252 (2)	0.19377 (15)	0.79093 (16)	0.0700 (5)	
H16	0.2842	0.1056	0.8185	0.084*	
C17	0.4432 (2)	0.22055 (14)	0.70430 (15)	0.0648 (4)	
H17	0.4825	0.1496	0.6736	0.078*	
C18	0.50517 (19)	0.35175 (13)	0.66164 (12)	0.0513 (3)	
H18	0.5843	0.3684	0.6025	0.062*	
C19	0.68575 (16)	0.73197 (11)	0.54723 (9)	0.0412 (2)	
C20	0.82935 (16)	0.77223 (12)	0.46545 (9)	0.0411 (2)	
C21	0.92074 (19)	0.67363 (16)	0.42316 (11)	0.0535 (3)	
H21	0.8911	0.5823	0.4474	0.064*	
C22	1.0549 (2)	0.7115 (2)	0.34546 (13)	0.0680 (4)	
H22	1.1148	0.6456	0.3169	0.082*	
C23	1.1010 (2)	0.8468 (2)	0.30971 (13)	0.0704 (5)	
H23	1.1924	0.8716	0.2576	0.085*	
C24	1.0122 (2)	0.94469 (19)	0.35095 (13)	0.0668 (4)	
H24	1.0432	1.0358	0.3267	0.080*	
C25	0.87667 (19)	0.90801 (14)	0.42849 (11)	0.0538 (3)	
H25	0.8167	0.9747	0.4561	0.065*	

### Atomic displacement parameters (Å^2^)

**Table:**

	*U*^11^	*U*^22^	*U*^33^	*U*^12^	*U*^13^	*U*^23^
S1	0.0582 (2)	0.03341 (13)	0.04398 (17)	0.00401 (11)	0.00390 (13)	−0.00090 (10)
N1	0.0345 (5)	0.0579 (6)	0.0390 (5)	0.0047 (4)	−0.0036 (4)	−0.0039 (4)
N2	0.0388 (5)	0.0427 (5)	0.0474 (5)	0.0021 (4)	−0.0070 (4)	−0.0069 (4)
N3	0.0578 (6)	0.0322 (4)	0.0531 (6)	0.0008 (4)	0.0132 (5)	−0.0036 (4)
N4	0.0507 (6)	0.0375 (4)	0.0478 (6)	0.0013 (4)	0.0057 (5)	−0.0070 (4)
C1	0.0616 (17)	0.0673 (18)	0.077 (2)	−0.0016 (14)	−0.0272 (18)	0.0131 (17)
C2	0.0502 (14)	0.0530 (13)	0.0565 (19)	0.0043 (11)	−0.0175 (15)	−0.0084 (13)
C1'	0.071 (4)	0.072 (4)	0.103 (6)	0.016 (3)	−0.015 (4)	0.002 (4)
C2'	0.056 (3)	0.074 (4)	0.047 (3)	0.008 (3)	−0.008 (2)	−0.018 (2)
C3	0.0351 (6)	0.0824 (10)	0.0485 (7)	0.0064 (6)	−0.0035 (5)	0.0055 (7)
C4	0.0419 (6)	0.0393 (5)	0.0373 (5)	−0.0035 (4)	−0.0028 (4)	−0.0028 (4)
C5	0.0560 (8)	0.0696 (9)	0.0488 (7)	−0.0062 (7)	−0.0127 (6)	−0.0113 (6)
C6	0.0862 (12)	0.0760 (10)	0.0476 (8)	−0.0061 (9)	−0.0109 (8)	−0.0226 (7)
C7	0.0810 (11)	0.0617 (8)	0.0523 (8)	0.0085 (8)	0.0023 (7)	−0.0215 (7)
C8	0.0535 (7)	0.0476 (6)	0.0539 (7)	0.0087 (5)	0.0015 (6)	−0.0106 (5)
C9	0.0413 (6)	0.0346 (5)	0.0412 (6)	−0.0003 (4)	−0.0016 (4)	−0.0043 (4)
C10	0.0432 (6)	0.0420 (5)	0.0447 (6)	0.0019 (4)	−0.0094 (5)	−0.0079 (4)
C11	0.0673 (10)	0.0751 (10)	0.0600 (9)	0.0064 (8)	−0.0238 (8)	−0.0249 (8)
C12	0.0429 (6)	0.0347 (4)	0.0386 (5)	0.0029 (4)	−0.0020 (4)	−0.0046 (4)
C13	0.0450 (6)	0.0344 (5)	0.0506 (7)	−0.0022 (4)	−0.0066 (5)	−0.0004 (4)
C14	0.0583 (8)	0.0463 (6)	0.0601 (8)	−0.0068 (6)	0.0047 (7)	0.0009 (6)
C15	0.0617 (9)	0.0574 (8)	0.0671 (9)	−0.0179 (7)	−0.0073 (8)	0.0125 (7)
C16	0.0749 (11)	0.0423 (6)	0.0889 (12)	−0.0179 (7)	−0.0306 (10)	0.0090 (7)
C17	0.0720 (10)	0.0383 (6)	0.0876 (12)	−0.0024 (6)	−0.0275 (9)	−0.0097 (7)
C18	0.0541 (7)	0.0392 (5)	0.0615 (8)	−0.0002 (5)	−0.0122 (6)	−0.0099 (5)
C19	0.0466 (6)	0.0377 (5)	0.0369 (5)	0.0017 (4)	−0.0022 (4)	−0.0051 (4)
C20	0.0414 (6)	0.0463 (6)	0.0342 (5)	−0.0002 (4)	−0.0053 (4)	−0.0053 (4)
C21	0.0511 (7)	0.0577 (7)	0.0533 (7)	0.0037 (6)	−0.0035 (6)	−0.0185 (6)
C22	0.0553 (9)	0.0885 (12)	0.0608 (9)	0.0084 (8)	0.0044 (7)	−0.0265 (9)
C23	0.0495 (8)	0.1031 (14)	0.0499 (8)	−0.0017 (8)	0.0073 (6)	−0.0061 (8)
C24	0.0571 (9)	0.0690 (9)	0.0609 (9)	−0.0085 (7)	0.0044 (7)	0.0082 (7)
C25	0.0539 (8)	0.0477 (6)	0.0519 (7)	−0.0015 (5)	0.0028 (6)	0.0000 (5)

### Geometric parameters (Å, °)

**Table:**

S1—C19	1.7585 (12)	C8—C9	1.3917 (17)
S1—C12	1.8872 (12)	C8—H8	0.9300
N1—C4	1.3863 (15)	C10—C11	1.4986 (19)
N1—C12	1.4263 (16)	C10—C12	1.5227 (17)
N1—C3	1.4692 (16)	C11—H11A	0.9600
N2—C10	1.2757 (16)	C11—H11B	0.9600
N2—C9	1.3991 (17)	C11—H11C	0.9600
N3—N4	1.3689 (15)	C13—C18	1.3887 (18)
N3—C13	1.4128 (15)	C13—C14	1.394 (2)
N3—C12	1.4709 (14)	C14—C15	1.391 (2)
N4—C19	1.2874 (15)	C14—H14	0.9300
C1—C2	1.318 (3)	C15—C16	1.367 (3)
C1—H1A	0.9300	C15—H15	0.9300
C1—H1B	0.9300	C16—C17	1.373 (3)
C2—C3	1.512 (3)	C16—H16	0.9300
C2—H2	0.9300	C17—C18	1.390 (2)
C1'—C2'	1.330 (5)	C17—H17	0.9300
C1'—H1'1	0.9300	C18—H18	0.9300
C1'—H1'2	0.9300	C19—C20	1.4629 (17)
C2'—C3	1.496 (4)	C20—C25	1.3896 (18)
C2'—H2'	0.9300	C20—C21	1.3948 (18)
C3—H3A	0.9700	C21—C22	1.378 (2)
C3—H3B	0.9700	C21—H21	0.9300
C4—C5	1.3975 (19)	C22—C23	1.381 (3)
C4—C9	1.4025 (17)	C22—H22	0.9300
C5—C6	1.381 (2)	C23—C24	1.374 (3)
C5—H5	0.9300	C23—H23	0.9300
C6—C7	1.377 (3)	C24—C25	1.382 (2)
C6—H6	0.9300	C24—H24	0.9300
C7—C8	1.373 (2)	C25—H25	0.9300
C7—H7	0.9300		
			
C19—S1—C12	89.97 (5)	C10—C11—H11A	109.5
C4—N1—C12	120.78 (10)	C10—C11—H11B	109.5
C4—N1—C3	120.12 (11)	H11A—C11—H11B	109.5
C12—N1—C3	117.17 (10)	C10—C11—H11C	109.5
C10—N2—C9	119.02 (11)	H11A—C11—H11C	109.5
N4—N3—C13	117.81 (10)	H11B—C11—H11C	109.5
N4—N3—C12	118.38 (9)	N1—C12—N3	111.72 (10)
C13—N3—C12	123.41 (10)	N1—C12—C10	112.44 (10)
C19—N4—N3	112.75 (10)	N3—C12—C10	111.80 (10)
C2—C1—H1A	120.0	N1—C12—S1	112.47 (8)
C2—C1—H1B	120.0	N3—C12—S1	100.87 (7)
H1A—C1—H1B	120.0	C10—C12—S1	106.89 (8)
C1—C2—C3	123.1 (3)	C18—C13—C14	119.45 (12)
C1—C2—H2	118.5	C18—C13—N3	119.04 (12)
C3—C2—H2	118.5	C14—C13—N3	121.50 (12)
C2'—C1'—H1'1	120.0	C15—C14—C13	119.39 (15)
C2'—C1'—H1'2	120.0	C15—C14—H14	120.3
H1'1—C1'—H1'2	120.0	C13—C14—H14	120.3
C1'—C2'—C3	114.3 (7)	C16—C15—C14	121.27 (17)
C1'—C2'—H2'	122.9	C16—C15—H15	119.4
C3—C2'—H2'	122.9	C14—C15—H15	119.4
N1—C3—C2'	113.4 (4)	C15—C16—C17	119.15 (14)
N1—C3—C2	112.4 (2)	C15—C16—H16	120.4
N1—C3—H3A	109.1	C17—C16—H16	120.4
C2'—C3—H3A	92.9	C16—C17—C18	121.24 (16)
C2—C3—H3A	109.1	C16—C17—H17	119.4
N1—C3—H3B	109.1	C18—C17—H17	119.4
C2'—C3—H3B	122.5	C13—C18—C17	119.49 (15)
C2—C3—H3B	109.1	C13—C18—H18	120.3
H3A—C3—H3B	107.9	C17—C18—H18	120.3
N1—C4—C5	122.78 (12)	N4—C19—C20	122.02 (11)
N1—C4—C9	118.77 (11)	N4—C19—S1	115.81 (9)
C5—C4—C9	118.41 (12)	C20—C19—S1	122.17 (9)
C6—C5—C4	119.98 (15)	C25—C20—C21	119.00 (12)
C6—C5—H5	120.0	C25—C20—C19	121.04 (11)
C4—C5—H5	120.0	C21—C20—C19	119.96 (11)
C7—C6—C5	121.61 (15)	C22—C21—C20	120.01 (15)
C7—C6—H6	119.2	C22—C21—H21	120.0
C5—C6—H6	119.2	C20—C21—H21	120.0
C8—C7—C6	118.93 (14)	C21—C22—C23	120.40 (16)
C8—C7—H7	120.5	C21—C22—H22	119.8
C6—C7—H7	120.5	C23—C22—H22	119.8
C7—C8—C9	120.97 (14)	C24—C23—C22	120.08 (15)
C7—C8—H8	119.5	C24—C23—H23	120.0
C9—C8—H8	119.5	C22—C23—H23	120.0
C8—C9—N2	117.88 (12)	C23—C24—C25	120.06 (16)
C8—C9—C4	120.09 (12)	C23—C24—H24	120.0
N2—C9—C4	121.98 (11)	C25—C24—H24	120.0
N2—C10—C11	119.02 (12)	C24—C25—C20	120.45 (14)
N2—C10—C12	123.99 (11)	C24—C25—H25	119.8
C11—C10—C12	116.96 (12)	C20—C25—H25	119.8
			
C13—N3—N4—C19	176.13 (12)	C13—N3—C12—S1	−171.95 (12)
C12—N3—N4—C19	−10.90 (18)	N2—C10—C12—N1	15.62 (17)
C4—N1—C3—C2'	67.3 (4)	C11—C10—C12—N1	−166.40 (12)
C12—N1—C3—C2'	−128.4 (4)	N2—C10—C12—N3	142.24 (12)
C4—N1—C3—C2	86.4 (3)	C11—C10—C12—N3	−39.79 (15)
C12—N1—C3—C2	−109.3 (3)	N2—C10—C12—S1	−108.26 (12)
C1'—C2'—C3—N1	144.0 (11)	C11—C10—C12—S1	69.71 (13)
C1'—C2'—C3—C2	53.6 (12)	C19—S1—C12—N1	106.99 (9)
C1—C2—C3—N1	−147.7 (7)	C19—S1—C12—N3	−12.17 (9)
C12—N1—C4—C5	−168.30 (12)	C19—S1—C12—C10	−129.14 (9)
C3—N1—C4—C5	−4.53 (19)	N4—N3—C13—C18	−11.6 (2)
C12—N1—C4—C9	13.84 (17)	C12—N3—C13—C18	175.83 (12)
C3—N1—C4—C9	177.61 (11)	N4—N3—C13—C14	169.30 (14)
N1—C4—C5—C6	−177.99 (14)	C12—N3—C13—C14	−3.3 (2)
C9—C4—C5—C6	−0.1 (2)	C18—C13—C14—C15	−0.9 (2)
C4—C5—C6—C7	0.5 (3)	N3—C13—C14—C15	178.20 (15)
C5—C6—C7—C8	−0.4 (3)	C13—C14—C15—C16	1.2 (3)
C6—C7—C8—C9	0.0 (2)	C14—C15—C16—C17	−0.5 (3)
C7—C8—C9—N2	177.98 (13)	C15—C16—C17—C18	−0.4 (3)
C7—C8—C9—C4	0.4 (2)	C14—C13—C18—C17	0.0 (2)
C10—N2—C9—C8	177.11 (12)	N3—C13—C18—C17	−179.11 (14)
C10—N2—C9—C4	−5.33 (17)	C16—C17—C18—C13	0.6 (2)
N1—C4—C9—C8	177.66 (11)	N3—N4—C19—C20	178.86 (11)
C5—C4—C9—C8	−0.29 (18)	N3—N4—C19—S1	−0.79 (16)
N1—C4—C9—N2	0.16 (17)	C12—S1—C19—N4	8.47 (11)
C5—C4—C9—N2	−177.80 (12)	C12—S1—C19—C20	−171.19 (11)
C9—N2—C10—C11	178.88 (12)	N4—C19—C20—C25	−175.82 (13)
C9—N2—C10—C12	−3.19 (18)	S1—C19—C20—C25	3.81 (18)
C4—N1—C12—N3	−147.06 (11)	N4—C19—C20—C21	4.4 (2)
C3—N1—C12—N3	48.70 (15)	S1—C19—C20—C21	−175.98 (10)
C4—N1—C12—C10	−20.40 (15)	C25—C20—C21—C22	−0.5 (2)
C3—N1—C12—C10	175.36 (11)	C19—C20—C21—C22	179.35 (14)
C4—N1—C12—S1	100.32 (11)	C20—C21—C22—C23	0.7 (3)
C3—N1—C12—S1	−63.91 (13)	C21—C22—C23—C24	−0.6 (3)
N4—N3—C12—N1	−104.20 (13)	C22—C23—C24—C25	0.2 (3)
C13—N3—C12—N1	68.34 (16)	C23—C24—C25—C20	0.1 (3)
N4—N3—C12—C10	128.80 (13)	C21—C20—C25—C24	0.0 (2)
C13—N3—C12—C10	−58.66 (16)	C19—C20—C25—C24	−179.75 (14)
N4—N3—C12—S1	15.50 (14)		
